# Supplementary material for: Effect of Bacterial Extracellular Polymeric Substances from Enterobacter spp. on Rice Growth under Abiotic Stress and Transcriptomic Analysis
Source: Microorganisms. 2024 Jun 16;12(6):1212. doi: 10.3390/microorganisms12061212 (PMC11205796; doi:10.3390/microorganisms12061212)
Supplement: Supplementary file 1 [file microorganisms-12-01212-s001.zip › Suplementary Figures.pdf]

Suplementary Materials

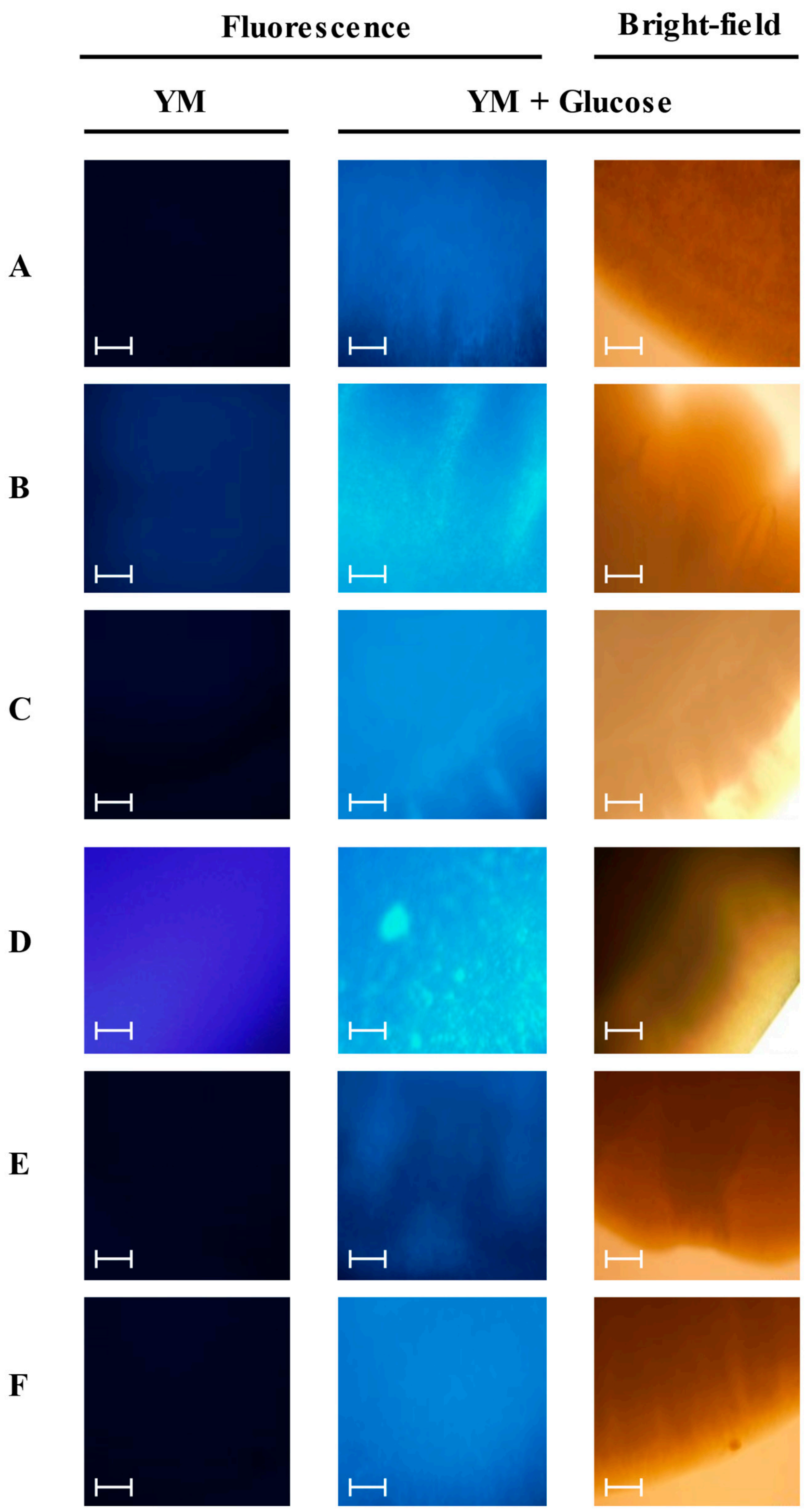

Figure S1: Fluorescent microscopic visualization of EPS matrix of isolates (A) JM160, (B) JW191, (C) JM187, (D) JC20, (E) JO32, and (F) JM63 in YM agar without and with 3%(w/v) glucose, stained with 1% of FB28 and observed with Olympus DP72 UplanApo 40X/0,85 immersion lens. The bright-field observation indicates the same area as with fluorescence. The scale bars indicate 200 µm.

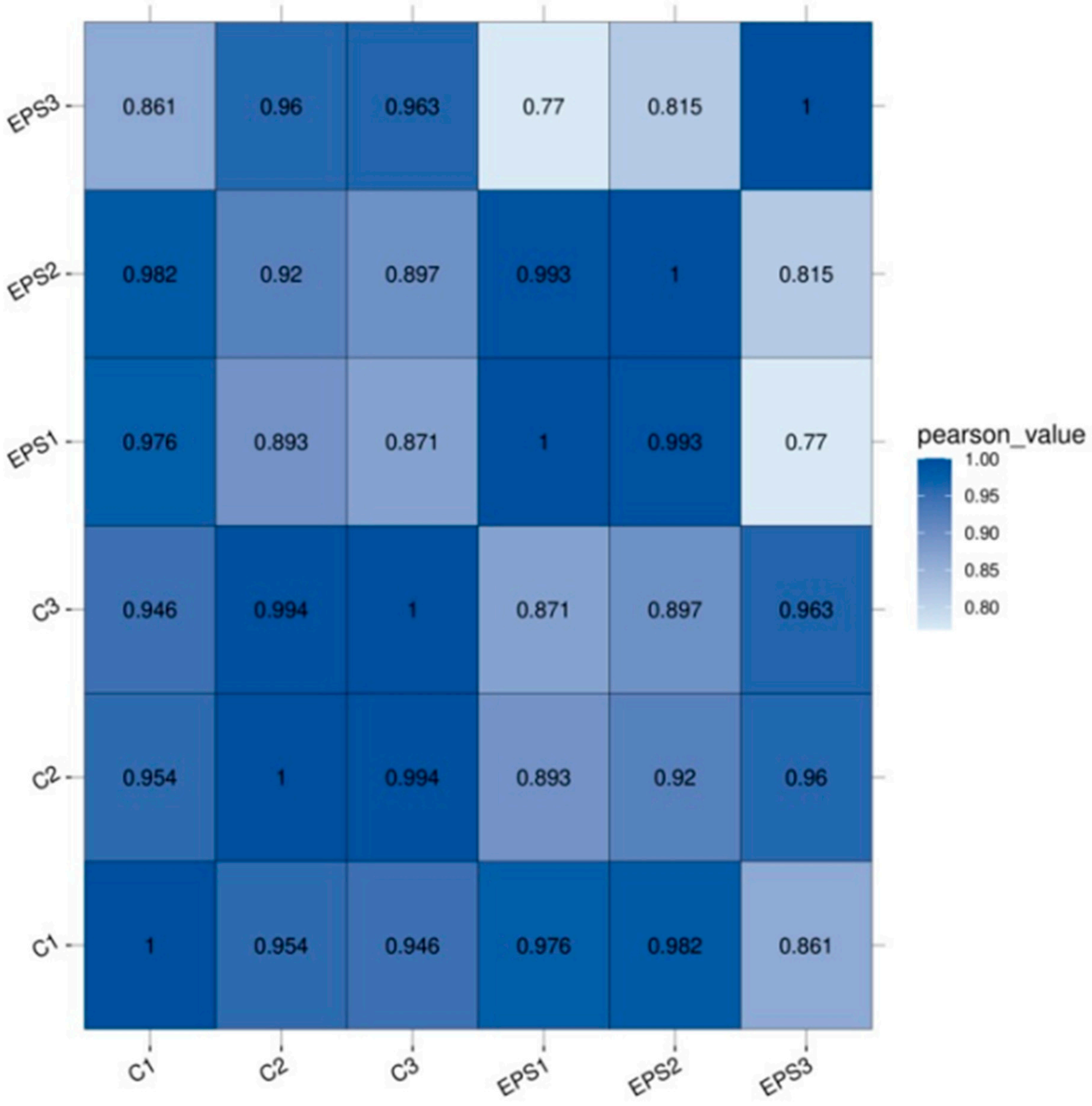

Figure S2: Samples correlation matrix. C: control seed samples; EPS: seed samples treated with Extracellular polymeric substances.

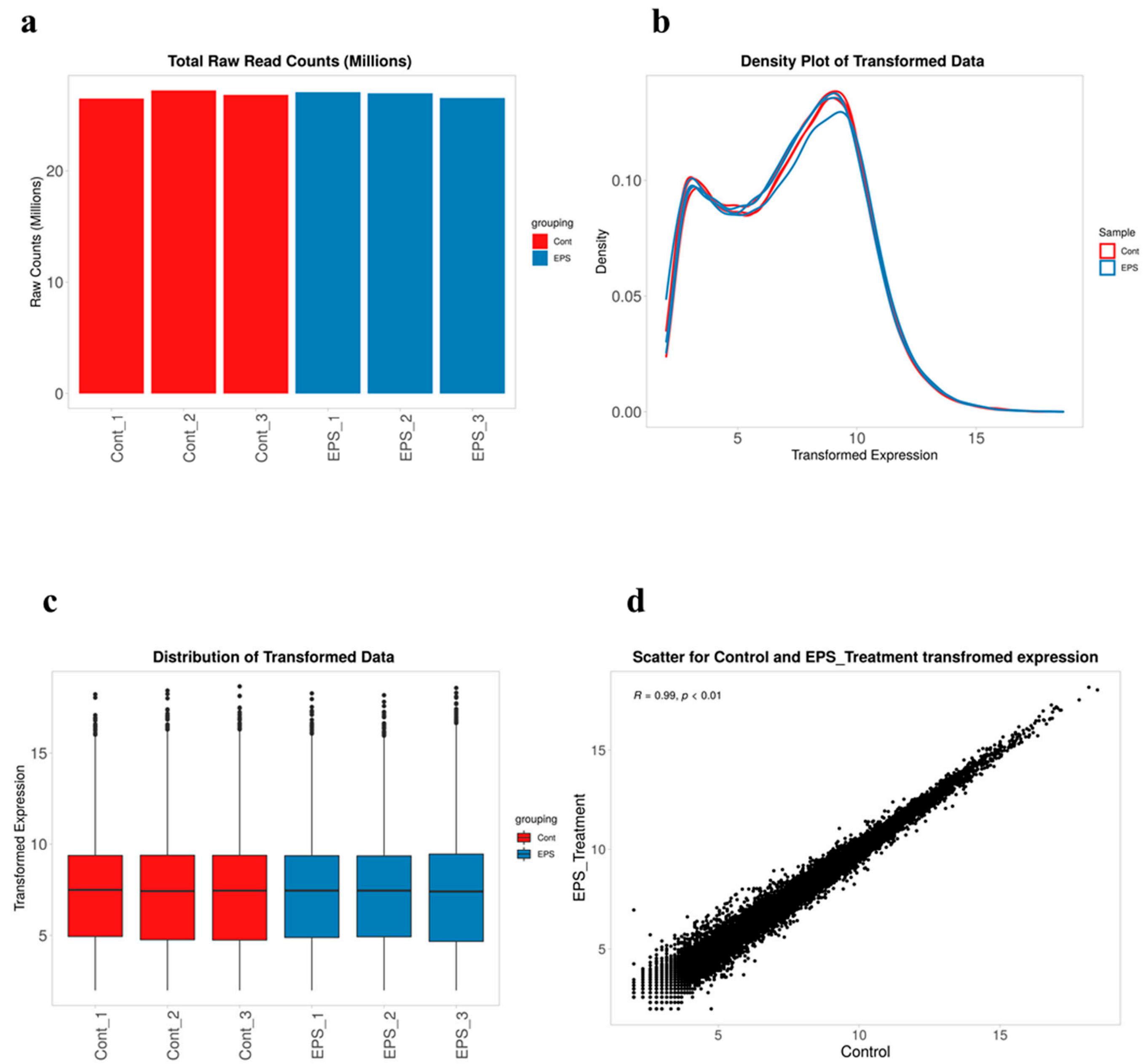

Figure S3: Diagnostic plots for read-counts data. (a). Total read counts per library. (b). Distribution of transformed data using a density plot. (c). Boxplot of transformed data. (d). Scatter plot of control and EPS treatment transformed expression. Cont: control seeds treated with water; EPS: seed samples treated with EPS.

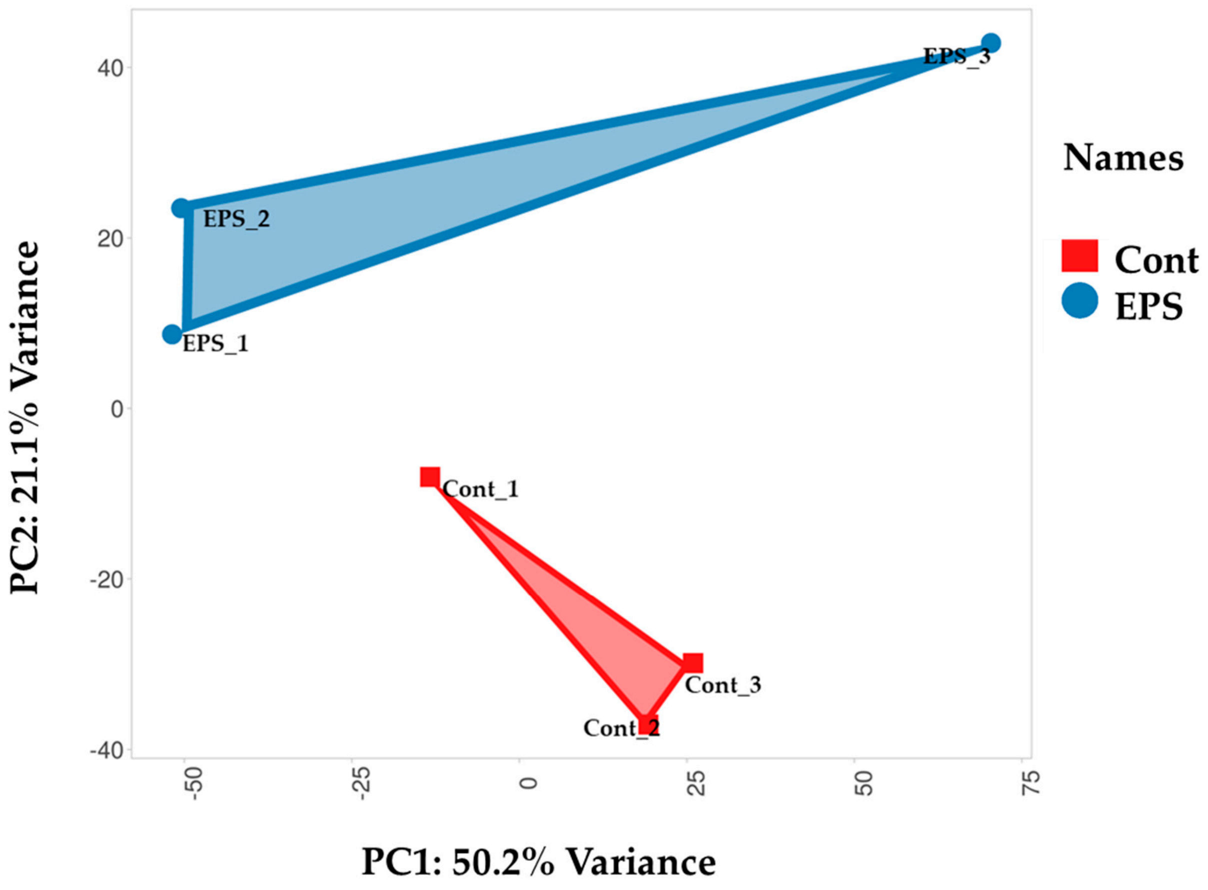

Figure S4: Principal Component Analyses (PCA). Cont: control seeds treated with water; EPS: seed samples treated with EPS. The samples in each group were triplicated.

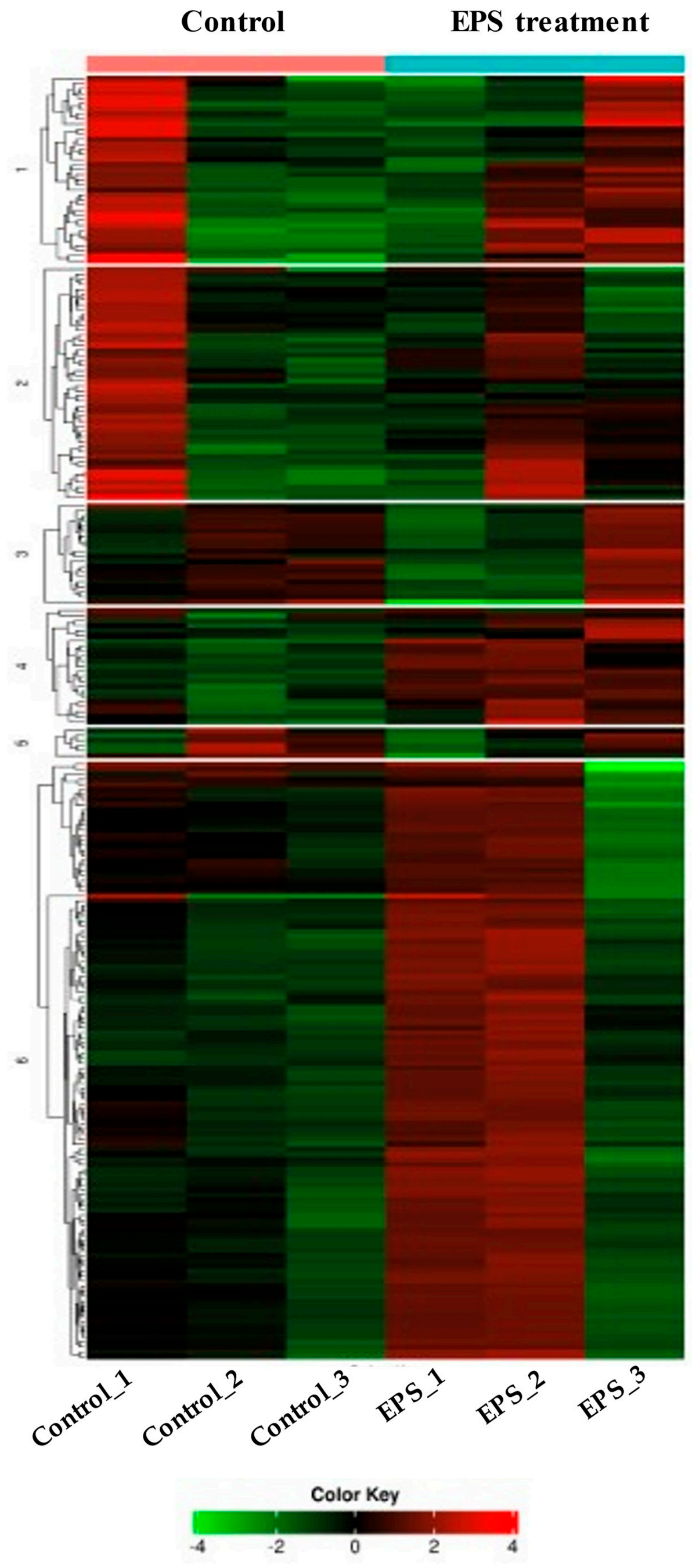

Figure S5: K-mean clustering

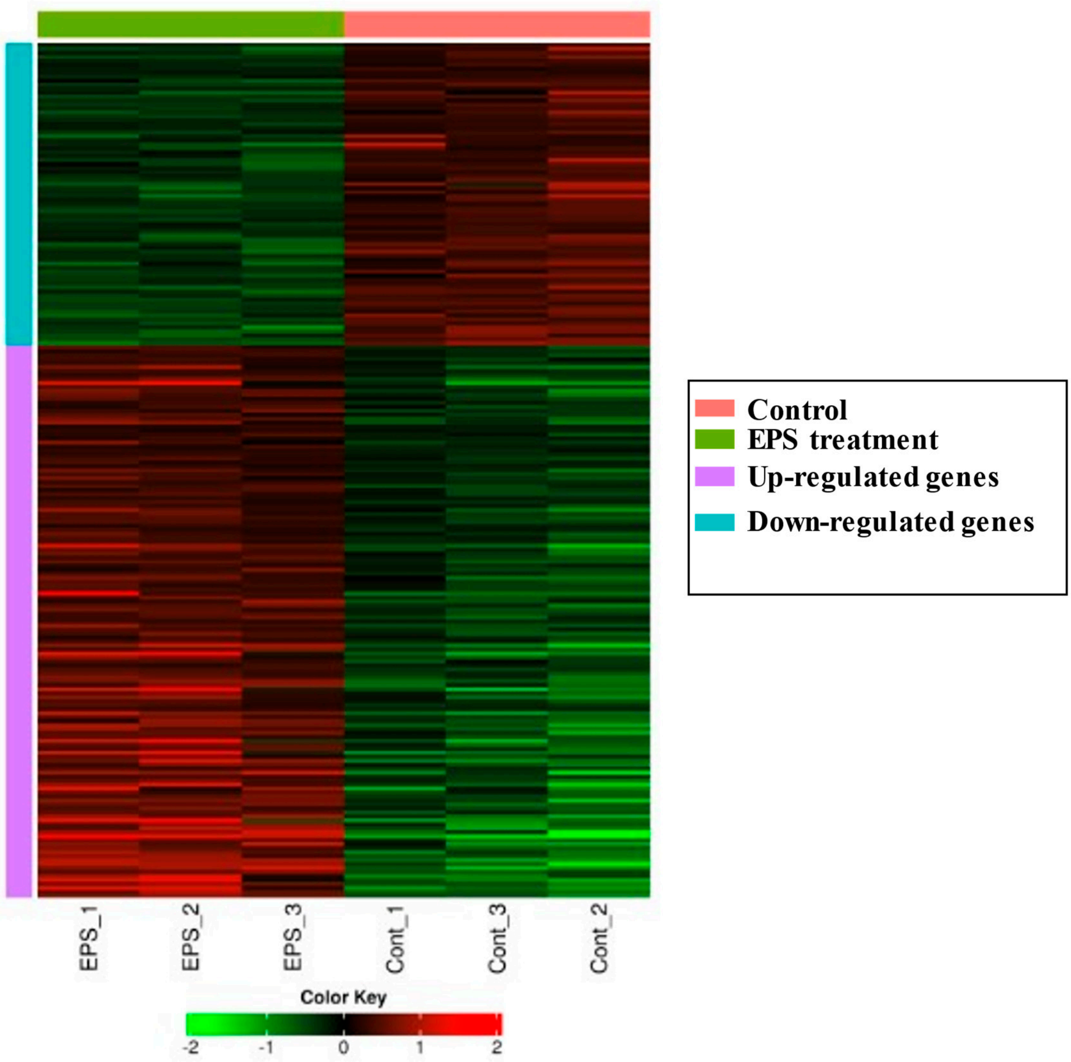

Figure S6: Heatmap (EPS vs Control) showing all associated DEGs (Sorted by FDR cutoff ≤0.05 and min fold-change=1). Each line represents one gene; the red color means a higher expression value with a positive fold change, while the green color means a lower expression value with a negative fold change.

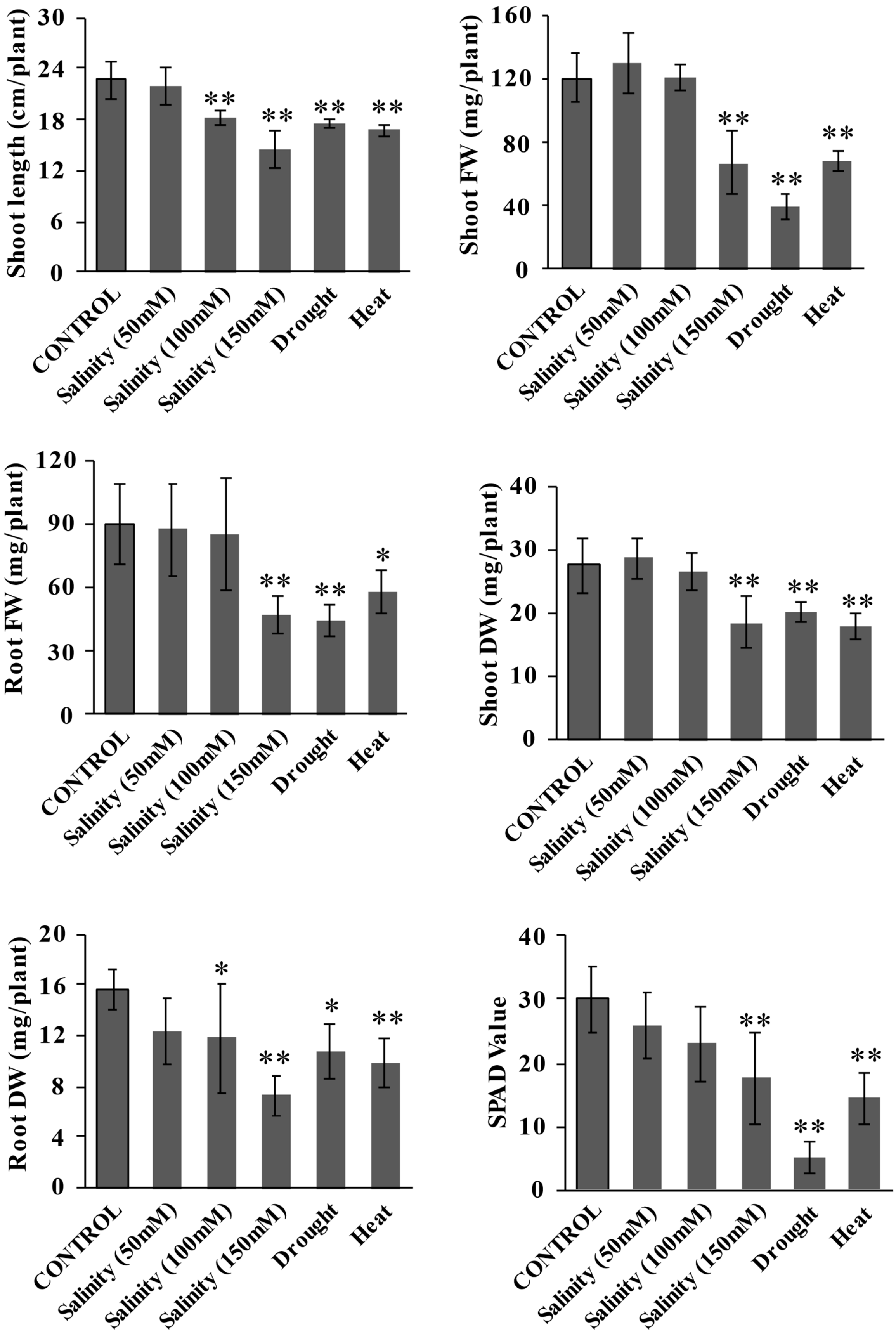

Figure S7: Effects of Abiotic stresses on rice growth (Dunnett's test one-way). Values are the means ± SD of five biological replicates. *: p ≤0.05; **: p ≤ 0.01.
