# Supplementary material for: Effect of Bacterial Extracellular Polymeric Substances from Enterobacter spp. on Rice Growth under Abiotic Stress and Transcriptomic Analysis
Source: Microorganisms. 2024 Jun 16;12(6):1212. doi: 10.3390/microorganisms12061212 (PMC11205796; doi:10.3390/microorganisms12061212)
Supplement: Supplementary file 1 [file microorganisms-12-01212-s001.zip › Suplementary Tables.pdf]

Supplementary Table S1. Primers used for RT-PCR

Primers	Sequences(5’-3’)	References
Ubiquitin_F	CACAAGAAGGTGAAGCTCGC	Ichimaru et al, 2022
Ubiquitin_R	GCCTTCTGGTTGTAGACGTAGG	
OsAAO5 F	ACGAAACCGATCATTTCCAAGCC	This study
*OsAAO5*_R	AAGCTGACGATCGAGCTCAAGG	

Supplementary Table S2. Reads filtering and Quality statistics after RNA seq

Sample	N read Num	Clean Read Num (10^6)	Total Clean Read	Total Mapping Genome Ratio (%)	Total Mapping Gene Ratio (%)	Uniquely Mapping Genome Ratio (%)	Uniquely Mapping Gene Ratio (%)	Gene expression density map (FPKM 1~10)
Control	98175	68.13	68.13	98.07	83.78	94.35	74.56	10519
EPS Treatment	97163	68	68.00	97.99	83.52	94.25	74.10	10836

Supplementary Table S3: GO classification of upregulated DEGs in rice seeds by EPS inoculation (sorted by p-value cutoff (FDR) ≤0.05)

Category	Pathway/Term	GO ID	Gene count	Corresponding DEGs ID	Fold Enrichment	Enrichment FDR (p-value)
**GO_BP**	Small molecule metabolic process	GO:0044281	21	Os01g0662700 Os01g0685800 Os01g0711000 Os01g0865100 Os02g0105400 Os03g0389700 Os03g0603600 Os03g0699700 Os03g0700700 Os03g0720300 Os04g0498600 Os05g0553000 Os06g0215600 Os06g0561000 Os07g0181000 Os09g0465600 Os10g0159800 Os10g0419400 Os11g0210500 Os12g0520200 Os12g0625000	3,69	0,00015
	Cellular response to toxic substance	GO:0097237	8	Os01g0326300 Os01g0963000 Os03g0368900 Os04g0688300 Os05g0134400 Os10g0109300 Os10g0159800 Os11g0210500	9,51	0,00039
	Detoxification	GO:0098754	8	Os01g0326300 Os01g0963000 Os03g0368900 Os04g0688300 Os05g0134400 Os10g0109300 Os10g0159800 Os11g0210500	9,33	0,00039
	Cellular detoxification	GO:1990748	8	Os01g0326300 Os01g0963000 Os03g0368900 Os04g0688300 Os05g0134400 Os10g0109300 Os10g0159800 Os11g0210500	9,51	0,00039
	Response to toxic substance	GO:0009636	8	Os01g0326300 Os01g0963000 Os03g0368900 Os04g0688300 Os05g0134400 Os10g0109300 Os10g0159800 Os11g0210500	9,01	0,00041
	Catabolic process	GO:0009056	21	Os01g0326300 Os01g0347600 Os01g0865100 Os01g0963000 Os02g0131400 Os03g0368900 Os03g0720300 Os04g0652700 Os04g0688300 Os05g0134400 Os06g0301300 Os06g0561000 Os07g0181000 Os07g0533300 Os07g0630400 Os09g0465600 Os10g0109300 Os10g0159800 Os10g0542900 Os11g0210500 Os12g0520200	3,09	0,00042
	Hydrogen peroxide catabolic process	GO:0042744	6	Os01g0326300 Os01g0963000 Os03g0368900 Os04g0688300 Os05g0134400 Os10g0109300	11,03	0,00146
	Small molecule biosynthetic process	GO:0044283	11	Os01g0662700 Os03g0389700 Os03g0699700 Os03g0700700 Os04g0498600 Os06g0215600 Os06g0561000 Os09g0465600 Os10g0419400 Os12g0520200 Os12g0625000	4,84	0,00146
	Hydrogen peroxide metabolic process	GO:0042743	6	Os01g0326300 Os01g0963000 Os03g0368900 Os04g0688300 Os05g0134400 Os10g0109300	10,65	0,00154
	Organic acid metabolic process	GO:0006082	13	Os01g0865100 Os02g0105400 Os03g0389700 Os03g0699700 Os03g0700700 Os03g0720300 Os06g0215600 Os06g0561000 Os07g0181000	3,73	0,00269

			Os09g0465600 Os10g0419400 Os12g0520200 Os12g0625000		
Cellular response to chemical stimulus	GO:0070887	12	Os01g0326300 Os01g0963000 Os03g0368900 Os04g0631100 Os04g0688300 Os05g0134400 Os07g0181000 Os10g0109300 Os10g0159800 Os11g0210500 Os12g0555000 Os12g0555200	3,98	0,00269
Purine-containing compound metabolic process	GO:0072521	7	Os01g0685800 Os01g0711000 Os01g0865100 Os04g0498600 Os05g0553000 Os07g0181000 Os09g0465600	7,39	0,00269
Reactive oxygen species metabolic process	GO:0072593	6	Os01g0326300 Os01g0963000 Os03g0368900 Os04g0688300 Os05g0134400 Os10g0109300	9,02	0,0027
Cellular catabolic process	GO:0044248	16	Os01g0326300 Os01g0347600 Os01g0963000 Os03g0368900 Os03g0720300 Os04g0652700 Os04g0688300 Os05g0134400 Os06g0301300 Os06g0561000 Os07g0630400 Os10g0109300 Os10g0159800 Os10g0542900 Os11g0210500 Os12g0520200	2,97	0,00433
Carboxylic acid metabolic process	GO:0019752	12	Os02g0105400 Os03g0389700 Os03g0699700 Os03g0700700 Os03g0720300 Os06g0215600 Os06g0561000 Os07g0181000 Os09g0465600 Os10g0419400 Os12g0520200 Os12g0625000	3,69	0,00447
Small molecule catabolic process	GO:0044282	6	Os01g0865100 Os03g0720300 Os06g0561000 Os10g0159800 Os11g0210500 Os12g0520200	7,74	0,00508
Oxoacid metabolic process	GO:0043436	12	Os02g0105400 Os03g0389700 Os03g0699700 Os03g0700700 Os03g0720300 Os06g0215600 Os06g0561000 Os07g0181000 Os09g0465600 Os10g0419400 Os12g0520200 Os12g0625000	3,56	0,00517
Cellular oxidant detoxification	GO:0098869	6	Os01g0326300 Os01g0963000 Os03g0368900 Os04g0688300 Os05g0134400 Os10g0109300	7,55	0,00517
Oxylipin metabolic process	GO:0031407	3	Os03g0699700 Os03g0700700 Os06g0215600	27,38	0,00537
Oxylipin biosynthetic process	GO:0031408	3	Os03g0699700 Os03g0700700 Os06g0215600	27,38	0,00537
Carboxylic acid biosynthetic process	GO:0046394	8	Os03g0389700 Os03g0699700 Os03g0700700 Os06g0215600 Os06g0561000 Os10g0419400 Os12g0520200 Os12g0625000	5,02	0,00607
Sulfur compound metabolic process	GO:0006790	7	Os01g0667200 Os04g0498600 Os07g0168300 Os07g0509800 Os10g0419400 Os10g0530900 Os12g0625000	5,64	0,00721
Organic acid biosynthetic process	GO:0016053	8	Os03g0389700 Os03g0699700 Os03g0700700 Os06g0215600 Os06g0561000 Os10g0419400 Os12g0520200 Os12g0625000	4,77	0,00788
ATP metabolic process	GO:0046034	5	Os01g0685800 Os01g0711000 Os05g0553000 Os07g0181000 Os09g0465600	8,3	0,00906

	ATP biosynthetic process	GO:0006754	3	Os01g0685800 Os01g0711000 Os05g0553000	20,72	0,0092
	Energy coupled proton transport, down electrochemical gradient	GO:0015985	3	Os01g0685800 Os01g0711000 Os05g0553000	20,72	0,0092
	ATP synthesis coupled proton transport	GO:0015986	3	Os01g0685800 Os01g0711000 Os05g0553000	20,72	0,0092
	Purine nucleoside triphosphate biosynthetic process	GO:0009145	3	Os01g0685800 Os01g0711000 Os05g0553000	17,83	0,01151
	Purine ribonucleoside triphosphate metabolic process	GO:0009205	3	Os01g0685800 Os01g0711000 Os05g0553000	17,83	0,01151
	Purine ribonucleoside triphosphate biosynthetic process	GO:0009206	3	Os01g0685800 Os01g0711000 Os05g0553000	17,83	0,01151
	Mitochondrial ATP synthesis coupled proton transport	GO:0042776	2	Os01g0685800 Os05g0553000	51,11	0,01151
	Formaldehyde metabolic process	GO:0046292	2	Os10g0159800 Os11g0210500	51,11	0,01151
	Formaldehyde catabolic process	GO:0046294	2	Os10g0159800 Os11g0210500	51,11	0,01151
	Cellular detoxification of aldehyde	GO:0110095	2	Os10g0159800 Os11g0210500	51,11	0,01151
	Cellular response to aldehyde	GO:0110096	2	Os10g0159800 Os11g0210500	51,11	0,01151
	Purine nucleoside triphosphate metabolic process	GO:0009144	3	Os01g0685800 Os01g0711000 Os05g0553000	17,42	0,01153
	Response to oxidative stress	GO:0006979	6	Os01g0326300 Os01g0963000 Os03g0368900 Os04g0688300 Os05g0134400 Os10g0109300	5,46	0,01354
	Nucleobase-containing small molecule metabolic process	GO:0055086	7	Os01g0685800 Os01g0711000 Os01g0865100 Os04g0498600 Os05g0553000 Os07g0181000 Os09g0465600	4,63	0,01354
	Purine-containing compound biosynthetic process	GO:0072522	4	Os01g0685800 Os01g0711000 Os04g0498600 Os05g0553000	9,46	0,01354
	Organic substance catabolic process	GO:1901575	15	Os01g0347600 Os01g0865100 Os02g0131400 Os03g0720300 Os04g0652700 Os06g0301300 Os06g0561000 Os07g0181000 Os07g0533300 Os07g0630400 Os09g0465600 Os10g0159800 Os10g0542900 Os11g0210500 Os12g0520200	2,51	0,01472

Ribonucleoside triphosphate metabolic process	GO:0009199	3	Os01g0685800 Os01g0711000 Os05g0553000	14,2	0,01799
Ribonucleoside triphosphate biosynthetic process	GO:0009201	3	Os01g0685800 Os01g0711000 Os05g0553000	14,2	0,01799
Nucleoside triphosphate biosynthetic process	GO:0009142	3	Os01g0685800 Os01g0711000 Os05g0553000	13,45	0,01935
Purine ribonucleotide metabolic process	GO:0009150	5	Os01g0685800 Os01g0711000 Os05g0553000 Os07g0181000 Os09g0465600	6,14	0,01935
Cellular response to oxygen-containing compound	GO:1901701	6	Os04g0631100 Os07g0181000 Os10g0159800 Os11g0210500 Os12g0555000 Os12g0555200	4,91	0,01935
Proton transmembrane transport	GO:1902600	4	Os01g0685800 Os01g0711000 Os05g0553000 Os07g0191200	8,31	0,01935
Purine nucleotide metabolic process	GO:0006163	5	Os01g0685800 Os01g0711000 Os05g0553000 Os07g0181000 Os09g0465600	5,86	0,02198
Generation of precursor metabolites and energy	GO:0006091	7	Os01g0501800 Os01g0685800 Os05g0553000 Os07g0181000 Os07g0695800 Os09g0346500 Os09g0465600	4,03	0,02366
Nucleoside triphosphate metabolic process	GO:0009141	3	Os01g0685800 Os01g0711000 Os05g0553000	12,17	0,02392
Aldehyde catabolic process	GO:0046185	2	Os10g0159800 Os11g0210500	30,06	0,02392
Ribonucleotide metabolic process	GO:0009259	5	Os01g0685800 Os01g0711000 Os05g0553000 Os07g0181000 Os09g0465600	5,48	0,02706
Carbohydrate metabolic process	GO:0005975	11	Os01g0660200 Os01g0713200 Os01g0946700 Os02g0105400 Os02g0131400 Os03g0603600 Os06g0561000 Os07g0181000 Os07g0539100 Os09g0465600 Os10g0542900	2,75	0,02713
Carbohydrate transport	GO:0008643	3	Os03g0168000 Os04g0452700 Os08g0535200	11,27	0,02768
Ribose phosphate metabolic process	GO:0019693	5	Os01g0685800 Os01g0711000 Os05g0553000 Os07g0181000 Os09g0465600	5,32	0,02903
Response to chemical	GO:0042221	12	Os01g0326300 Os01g0963000 Os03g0368900 Os04g0631100 Os04g0688300 Os05g0134400 Os07g0181000 Os10g0109300 Os10g0159800 Os11g0210500 Os12g0555000 Os12g0555200	2,54	0,03117
Regulation of protein serine/threonine phosphatase activity	GO:0080163	2	Os12g0555000 Os12g0555200	23,23	0,03581
Response to stress	GO:0006950	15	Os01g0326300 Os01g0667200 Os01g0963000 Os03g0133400 Os03g0368900 Os04g0631100 Os04g0688300 Os05g0134400 Os06g0301300	2,13	0,04869

				Os08g0535200 Os10g0109300 Os10g0191300 Os10g0542900 Os12g0437800 Os12g0555200		
**GO_MF**	Oxidoreductase activity	GO:0016491	30	Os01g0127000 Os01g0326300 Os01g0501800 Os01g0667200 Os01g0723400 Os01g0784800 Os01g0865100 Os01g0878900 Os01g0963000 Os02g0105400 Os03g0368900 Os03g0699700 Os03g0700700 Os04g0179200 Os04g0688300 Os05g0134400 Os06g0215600 Os06g0338200 Os06g0549900 Os06g0561000 Os07g0418500 Os07g0509800 Os07g0695800 Os08g0557600 Os09g0507300 Os10g0109300 Os10g0159800 Os10g0419400 Os10g0496900 Os11g0210500	4,26	0,00000
	Oxidoreductase activity, acting on single donors with incorporation of molecular oxygen	GO:0016701	6	Os01g0667200 Os01g0878900 Os03g0699700 Os03g0700700 Os06g0561000 Os10g0419400	24,34	0,00003
	Cation binding	GO:0043169	30	Os01g0127000 Os01g0326300 Os01g0723400 Os01g0878900 Os01g0963000 Os03g0368900 Os03g0699700 Os03g0700700 Os03g0804500 Os04g0288100 Os04g0652700 Os04g0688300 Os05g0134400 Os06g0338200 Os06g0561000 Os07g0181000 Os07g0418500 Os07g0695800 Os08g0189600 Os09g0346500 Os09g0507300 Os10g0109300 Os10g0159800 Os10g0419400 Os11g0210500 Os11g0528500 Os11g0600700 Os12g0154700 Os12g0154800 Os12g0637100	2,49	0,00023
	Manganese ion binding	GO:0030145	5	Os03g0804500 Os04g0288100 Os08g0189600 Os12g0154700 Os12g0154800	20,61	0,00032
	Metal ion binding	GO:0046872	29	Os01g0127000 Os01g0326300 Os01g0723400 Os01g0878900 Os01g0963000 Os03g0368900 Os03g0699700 Os03g0700700 Os03g0804500 Os04g0288100 Os04g0652700 Os04g0688300 Os05g0134400 Os06g0338200 Os06g0561000 Os07g0181000 Os07g0418500 Os08g0189600 Os09g0346500 Os09g0507300 Os10g0109300 Os10g0159800 Os10g0419400 Os11g0210500 Os11g0528500 Os11g0600700 Os12g0154700 Os12g0154800 Os12g0637100	2,43	0,00033
	Transition metal ion binding	GO:0046914	16	Os01g0127000 Os01g0878900 Os03g0804500 Os04g0288100 Os06g0338200 Os06g0561000 Os07g0418500 Os08g0189600 Os09g0507300 Os10g0159800 Os10g0419400 Os11g0210500	3,43	0,00085

			Os11g0528500 Os11g0600700 Os12g0154700 Os12g0154800		
Pyrophosphate hydrolysis-driven proton transmembrane transporter activity	GO:0009678	4	Os01g0685800 Os01g0711000 Os05g0553000 Os07g0191200	20,86	0,00141
Clathrin light chain binding	GO:0032051	2	Os11g0104866 Os12g0104766	170,36	0,00141
Nutrient reservoir activity	GO:0045735	5	Os03g0804500 Os04g0288100 Os08g0189600 Os12g0154700 Os12g0154800	13,04	0,00141
Oxidoreductase activity, acting on single donors with incorporation of molecular oxygen, incorporation of two atoms of oxygen	GO:0016702	4	Os01g0667200 Os03g0699700 Os03g0700700 Os10g0419400	19,29	0,00158
Peroxidase activity	GO:0004601	6	Os01g0326300 Os01g0963000 Os03g0368900 Os04g0688300 Os05g0134400 Os10g0109300	8,61	0,00169
Proton channel activity	GO:0015252	3	Os01g0685800 Os01g0711000 Os05g0553000	34,85	0,00169
Oxidoreductase activity, acting on peroxide as acceptor	GO:0016684	6	Os01g0326300 Os01g0963000 Os03g0368900 Os04g0688300 Os05g0134400 Os10g0109300	8,57	0,00169
Proton-transporting ATP synthase activity, rotational mechanism	GO:0046933	3	Os01g0685800 Os01g0711000 Os05g0553000	34,85	0,00169
Linoleate 9S-lipoxygenase activity	GO:1990136	2	Os03g0699700 Os03g0700700	127,77	0,00169
ATPase-coupled cation transmembrane transporter activity	GO:0019829	4	Os01g0685800 Os01g0711000 Os05g0553000 Os07g0191200	15,73	0,00186
ATPase-coupled ion transmembrane transporter activity	GO:0042625	3	Os01g0685800 Os01g0711000 Os05g0553000	30,66	0,00186
ATPase activity, coupled to transmembrane movement of ions, rotational mechanism	GO:0044769	3	Os01g0685800 Os01g0711000 Os05g0553000	30,66	0,00186
Proton-transporting ATPase activity, rotational mechanism	GO:0046961	3	Os01g0685800 Os01g0711000 Os05g0553000	30,66	0,00186
Antioxidant activity	GO:0016209	6	Os01g0326300 Os01g0963000 Os03g0368900 Os04g0688300 Os05g0134400 Os10g0109300	7,44	0,00234
Alcohol dehydrogenase activity, zinc-dependent	GO:0004024	2	Os10g0159800 Os11g0210500	56,79	0,00682

	S-(hydroxymethyl)glutathione dehydrogenase activity	GO:0051903	2	Os10g0159800 Os11g0210500	56,79	0,00682
	Alcohol dehydrogenase (NAD+) activity	GO:0004022	2	Os10g0159800 Os11g0210500	51,11	0,00779
	Alcohol dehydrogenase [NAD(P)+] activity	GO:0018455	2	Os10g0159800 Os11g0210500	51,11	0,00779
	Dioxygenase activity	GO:0051213	5	Os01g0667200 Os01g0878900 Os03g0699700 Os03g0700700 Os10g0419400	7,18	0,00780
	Oxidoreductase activity, acting on the CH-OH group of donors, NAD or NADP as acceptor	GO:0016616	5	Os01g0723400 Os02g0105400 Os04g0179200 Os10g0159800 Os11g0210500	5,86	0,01843
	Tetrapyrrole binding	GO:0046906	8	Os01g0326300 Os01g0963000 Os03g0368900 Os04g0688300 Os05g0134400 Os07g0418500 Os09g0346500 Os10g0109300	3,62	0,01843
	Cation channel activity	GO:0005261	3	Os01g0685800 Os01g0711000 Os05g0553000	12,17	0,01955
	Active transmembrane transporter activity	GO:0022804	8	Os01g0685800 Os01g0711000 Os03g0168000 Os04g0452700 Os04g0631100 Os05g0553000 Os07g0191200 Os10g0580400	3,41	0,02486
	Oxidoreductase activity, acting on CH-OH group of donors	GO:0016614	5	Os01g0723400 Os02g0105400 Os04g0179200 Os10g0159800 Os11g0210500	5,11	0,02889
	Abscisic acid binding	GO:0010427	2	Os12g0555000 Os12g0555200	21,29	0,03570
	Transferase activity, transferring alkyl or aryl (other than methyl) groups	GO:0016765	4	Os03g0389700 Os07g0168300 Os10g0530900 Os12g0625000	6,19	0,03586
	Isoprenoid binding	GO:0019840	2	Os12g0555000 Os12g0555200	20,44	0,03636
	Proton transmembrane transporter activity	GO:0015078	5	Os01g0685800 Os01g0711000 Os04g0452700 Os05g0553000 Os07g0191200	4,65	0,03815
	L-lactate dehydrogenase activity	GO:0004459	1	Os02g0105400	127,77	0,04370
	Hydrolase activity, hydrolyzing O-glycosyl compounds	GO:0004553	6	Os01g0660200 Os01g0713200 Os01g0946700 Os02g0131400 Os07g0539100 Os10g0542900	3,76	0,04370
	Oxoglutarate dehydrogenase (succinyl-transferring) activity	GO:0004591	1	Os07g0695800	127,77	0,04370
	Protein phosphatase inhibitor activity	GO:0004864	2	Os12g0555000 Os12g0555200	16,49	0,04370
	Copper ion binding	GO:0005507	3	Os01g0127000 Os06g0338200 Os09g0507300	7,99	0,04370
	Oxygen evolving activity	GO:0010242	1	Os01g0501800	127,77	0,04370

	Symporter activity	GO:0015293	4	Os03g0168000 Os04g0452700 Os04g0631100 Os10g0580400	5,59	0,04370
	Peptidase activator activity	GO:0016504	1	Os06g0301300	127,77	0,04370
	Protochlorophyllide reductase activity	GO:0016630	1	Os10g0496900	127,77	0,04370
	Phosphatase inhibitor activity	GO:0019212	2	Os12g0555000 Os12g0555200	15,97	0,04370
	Heme binding	GO:0020037	7	Os01g0326300 Os01g0963000 Os03g0368900 Os04g0688300 Os05g0134400 Os07g0418500 Os10g0109300	3,28	0,04370
	Active ion transmembrane transporter activity	GO:0022853	5	Os01g0685800 Os01g0711000 Os04g0452700 Os05g0553000 Os07g0191200	4,1	0,04370
	Histone kinase activity	GO:0035173	1	Os01g0191800	127,77	0,04370
	Histone serine kinase activity	GO:0035174	1	Os01g0191800	127,77	0,04370
	Hormone binding	GO:0042562	2	Os12g0555000 Os12g0555200	16,49	0,04370
	ATPase-coupled transmembrane transporter activity	GO:0042626	4	Os01g0685800 Os01g0711000 Os05g0553000 Os07g0191200	5,47	0,04370
	Transporter activity	GO:0005215	12	Os01g0685800 Os01g0711000 Os03g0168000 Os03g0838400 Os04g0452700 Os04g0631100 Os05g0553000 Os06g0708700 Os07g0191200 Os08g0535200 Os08g0556300 Os10g0580400	2,22	0,04376
	FMN binding	GO:0010181	2	Os01g0784800 Os06g0215600	14,6	0,04376
	Alcohol binding	GO:0043178	2	Os12g0555000 Os12g0555200	14,6	0,04376
**GO_CC**	Extracellular region	GO:0005576	18	Os01g0326300 Os01g0347600 Os01g0660200 Os01g0963000 Os02g0131400 Os03g0368900 Os03g0804500 Os04g0288100 Os04g0688300 Os05g0134400 Os07g0533300 Os07g0630400 Os08g0189600 Os09g0507300 Os10g0109300 Os10g0191300 Os12g0154700 Os12g0154800	5,58	0
	Cell periphery	GO:0071944	28	Os01g0326300 Os01g0713200 Os01g0946700 Os01g0963000 Os02g0807900 Os03g0133400 Os03g0168000 Os03g0368900 Os03g0804500 Os03g0838400 Os04g0288100 Os04g0452700 Os04g0688300 Os05g0134400 Os06g0708700 Os07g0191200 Os07g0442900 Os07g0539100 Os08g0189600 Os08g0535200 Os09g0551500 Os10g0422075 Os10g0580400 Os11g0104866 Os11g0514500 Os12g0104766 Os12g0154700 Os12g0154800	2,85	0,00003

Cell wall	GO:0005618	10	Os01g0326300 Os01g0963000 Os03g0368900 Os03g0804500 Os04g0288100 Os04g0688300 Os05g0134400 Os08g0189600 Os12g0154700 Os12g0154800	6,72	0,00011
External encapsulating structure	GO:0030312	10	Os01g0326300 Os01g0963000 Os03g0368900 Os03g0804500 Os04g0288100 Os04g0688300 Os05g0134400 Os08g0189600 Os12g0154700 Os12g0154800	6,59	0,00011
Proton-transporting ATP synthase complex, catalytic core F(1)	GO:0045261	3	Os01g0685800 Os01g0711000 Os05g0553000	42,59	0,00106
Clathrin complex	GO:0071439	2	Os11g0104866 Os12g0104766	170,36	0,00106
Cell-cell junction	GO:0005911	7	Os01g0326300 Os01g0963000 Os03g0368900 Os04g0688300 Os05g0134400 Os11g0104866 Os12g0104766	6,5	0,0014
Plasmodesma	GO:0009506	7	Os01g0326300 Os01g0963000 Os03g0368900 Os04g0688300 Os05g0134400 Os11g0104866 Os12g0104766	6,5	0,0014
Cell junction	GO:0030054	7	Os01g0326300 Os01g0963000 Os03g0368900 Os04g0688300 Os05g0134400 Os11g0104866 Os12g0104766	6,5	0,0014
Symplast	GO:0055044	7	Os01g0326300 Os01g0963000 Os03g0368900 Os04g0688300 Os05g0134400 Os11g0104866 Os12g0104766	6,5	0,0014
Anchoring junction	GO:0070161	7	Os01g0326300 Os01g0963000 Os03g0368900 Os04g0688300 Os05g0134400 Os11g0104866 Os12g0104766	6,5	0,0014
Proton-transporting two-sector ATPase complex, catalytic domain	GO:0033178	3	Os01g0685800 Os01g0711000 Os05g0553000	27,38	0,00207
Clathrin coat of coated pit	GO:0030132	2	Os11g0104866 Os12g0104766	85,18	0,00241
Proton-transporting ATP synthase complex	GO:0045259	3	Os01g0685800 Os01g0711000 Os05g0553000	24,73	0,00242
Mitochondrial proton-transporting ATP synthase complex, catalytic sector F(1)	GO:0000275	2	Os01g0685800 Os05g0553000	73,01	0,0027
Plant-type cell wall	GO:0009505	5	Os01g0326300 Os01g0963000 Os03g0368900 Os04g0688300 Os05g0134400	8,46	0,0027
Clathrin coat of trans-Golgi network vesicle	GO:0030130	2	Os11g0104866 Os12g0104766	73,01	0,0027
Trans-Golgi network transport vesicle membrane	GO:0012510	2	Os11g0104866 Os12g0104766	63,88	0,00307

Trans-Golgi network transport vesicle	GO:0030140	2	Os11g0104866 Os12g0104766	63,88	0,00307
Intrinsic component of plasma membrane	GO:0031226	7	Os01g0713200 Os01g0946700 Os03g0168000 Os03g0838400 Os04g0452700 Os07g0539100 Os08g0535200	5,15	0,00315
Thylakoid membrane	GO:0042651	6	Os01g0246400 Os01g0501800 Os01g0711000 Os04g0631100 Os07g0178800 Os09g0346500	5,68	0,00467
Photosynthetic membrane	GO:0034357	6	Os01g0246400 Os01g0501800 Os01g0711000 Os04g0631100 Os07g0178800 Os09g0346500	5,31	0,00633
Plasma membrane	GO:0005886	18	Os01g0713200 Os01g0946700 Os02g0807900 Os03g0133400 Os03g0168000 Os03g0838400 Os04g0452700 Os06g0708700 Os07g0191200 Os07g0442900 Os07g0539100 Os08g0535200 Os09g0551500 Os10g0422075 Os10g0580400 Os11g0104866 Os11g0514500 Os12g0104766	2,25	0,00651
Proton-transporting two-sector ATPase complex	GO:0016469	3	Os01g0685800 Os01g0711000 Os05g0553000	13,94	0,00768
Apoplast	GO:0048046	5	Os03g0804500 Os04g0288100 Os08g0189600 Os12g0154700 Os12g0154800	6,08	0,00811
Clathrin vesicle coat	GO:0030125	2	Os11g0104866 Os12g0104766	34,07	0,00825
Clathrin-coated vesicle membrane	GO:0030665	2	Os11g0104866 Os12g0104766	31,94	0,00906
Mitochondrial proton-transporting ATP synthase complex	GO:0005753	2	Os01g0685800 Os05g0553000	26,9	0,01153
Chloroplast thylakoid membrane	GO:0009535	5	Os01g0246400 Os01g0711000 Os04g0631100 Os07g0178800 Os09g0346500	5,41	0,01153
Plastid thylakoid membrane	GO:0055035	5	Os01g0246400 Os01g0711000 Os04g0631100 Os07g0178800 Os09g0346500	5,41	0,01153
Clathrin-coated pit	GO:0005905	2	Os11g0104866 Os12g0104766	24,34	0,01349
Thylakoid	GO:0009579	6	Os01g0246400 Os01g0501800 Os01g0711000 Os04g0631100 Os07g0178800 Os09g0346500	4,24	0,01349
Clathrin coat	GO:0030118	2	Os11g0104866 Os12g0104766	22,22	0,01535
Integral component of plasma membrane	GO:0005887	4	Os03g0168000 Os03g0838400 Os04g0452700 Os08g0535200	6,01	0,01869
Chloroplast thylakoid	GO:0009534	5	Os01g0246400 Os01g0711000 Os04g0631100 Os07g0178800 Os09g0346500	4,56	0,01935
Plastid thylakoid	GO:0031976	5	Os01g0246400 Os01g0711000 Os04g0631100 Os07g0178800 Os09g0346500	4,56	0,01935
Plasma membrane region	GO:0098590	2	Os11g0104866 Os12g0104766	17,04	0,02313
Clathrin-coated vesicle	GO:0030136	2	Os11g0104866 Os12g0104766	15,49	0,02643

Membrane protein complex	GO:0098796	7	Os01g0501800 Os01g0685800 Os01g0711000 Os05g0553000 Os09g0346500 Os11g0104866 Os12g0104766	3,14	0,02643
Chromosome passenger complex	GO:0032133	1	Os01g0191800	127,77	0,02714
Golgi-associated vesicle membrane	GO:0030660	2	Os11g0104866 Os12g0104766	14,6	0,02818
Chloroplast	GO:0009507	12	Os01g0246400 Os01g0711000 Os02g0115600 Os03g0389700 Os04g0631100 Os07g0178800 Os07g0181000 Os09g0346500 Os09g0465600 Os10g0496900 Os11g0104866 Os12g0104766	2,19	0,02903
Plasma membrane protein complex	GO:0098797	2	Os11g0104866 Os12g0104766	14,2	0,02903
Golgi-associated vesicle	GO:0005798	2	Os11g0104866 Os12g0104766	13,45	0,03079
Plastid	GO:0009536	12	Os01g0246400 Os01g0711000 Os02g0115600 Os03g0389700 Os04g0631100 Os07g0178800 Os07g0181000 Os09g0346500 Os09g0465600 Os10g0496900 Os11g0104866 Os12g0104766	2,13	0,03398
Transport vesicle	GO:0030133	2	Os11g0104866 Os12g0104766	10,87	0,04333
Transport vesicle membrane	GO:0030658	2	Os11g0104866 Os12g0104766	10,87	0,04333
Plastid membrane	GO:0042170	5	Os01g0246400 Os01g0711000 Os04g0631100 Os07g0178800 Os09g0346500	3,44	0,04522

FDR is adjusted from the hypergeometric test. Fold Enrichment is defined as the percentage of genes in our list belonging to a pathway, divided by the corresponding percentage in the background. FDR tells us how likely the enrichment is by chance; Fold Enrichment indicates how drastically genes of a certain pathway are overrepresented.

Supplementary Table S4: GO Classification of downregulated DEGs in rice seeds by EPS inoculation (sorted by p-value cutoff (FDR) ≤0.05)

GO category	Pathway/Term	GO ID	Gene count	Corresponding DEGs ID	Fold Enrichment	Enrichment FDR (p-value)
**GO_BP**	Amine transport	GO:0015837	2	Os06g0633100 Os06g0654400	207.39	0.00182
	Regulation of organic acid transport	GO:0032890	2	Os06g0633100 Os06g0654400	207.39	0.00182
	Amino acid export across plasma membrane	GO:0032973	2	Os06g0633100 Os06g0654400	207.39	0.00182
	Regulation of amine transport	GO:0051952	2	Os06g0633100 Os06g0654400	207.39	0.00182
	Regulation of amino acid transport	GO:0051955	2	Os06g0633100 Os06g0654400	207.39	0.00182
	Regulation of amino acid export	GO:0080143	2	Os06g0633100 Os06g0654400	207.39	0.00182
	Regulation of amino acid transmembrane transport	GO:1903789	2	Os06g0633100 Os06g0654400	207.39	0.00182
	Response to heat	GO:0009408	4	Os01g0184100 Os03g0245800 Os06g0592500 Os09g0526600	15.14	0.00638
	Export across plasma membrane	GO:0140115	2	Os06g0633100 Os06g0654400	74.07	0.01271
	Regulation of transmembrane transport	GO:0034762	2	Os06g0633100 Os06g0654400	51.85	0.02370
	Response to temperature stimulus	GO:0009266	4	Os01g0184100 Os03g0245800 Os06g0592500 Os09g0526600	8.50	0.04080

FDR is adjusted from the hypergeometric test. Fold Enrichment is defined as the percentage of genes in our gene list belonging to a pathway, divided by the corresponding percentage in the background. FDR tells us how likely the enrichment is by chance; Fold Enrichment indicates how drastically genes of a certain pathway are overrepresented.
